# The role of provider characteristics on the hepatitis A and B vaccination status of adults in the United States during 2007–2015

**DOI:** 10.1016/j.pmedr.2019.100833

**Published:** 2019-02-18

**Authors:** P.K. Ghaswalla, B.J. Patterson, W.Y. Cheng, E. Duchesneau, M. Macheca, M.S. Duh, A.F. Trofa

**Affiliations:** a GSK, 5 Crescent Drive, Philadelphia, PA 19112, USA; b Analysis Group, Inc., 111 Huntington Ave. 14th Floor, Boston, MA 02199, USA

**Keywords:** ACIP, advisory committee on immunization practices, CDC, centers for disease control and prevention, HBV, hepatitis B virus, CLD, chronic liver disease, CPT, current procedural terminology, EMR, electronic medical record, ICD-9-CM, international classification of disease, ninth revision, clinical modification, Adult, Hepatitis A, Hepatitis B, United States, Vaccination

## Abstract

Hepatitis A and B vaccine coverage is suboptimal in US adults, even among those at increased risk for infection, morbidity, or mortality. To understand where medical education and resources might enhance vaccine coverage, it is important to first identify providers and places most commonly associated with the administration of hepatitis vaccinations.

We conducted a retrospective analysis of commercial and Medicare insurance claims data from 2007 to 2015 to describe provider types and places of vaccination against hepatitis A and B among adults in the US, and estimated the time to initial vaccination from first diagnosis of a condition for which the Advisory Committee on Immunization Practices (ACIP) recommends hepatitis A and/or B vaccination among at-risk adults. We identified 183,326 adults who received hepatitis A vaccine, 148,119 hepatitis B vaccine, and 64,953 a bivalent vaccine. Mean age was 42.1–45.8 years. Family practice and internal medicine physicians were the main vaccine providers: 38.9% and 20.2% for hepatitis A, 43.7% and 21.4% for hepatitis B, 35.3% and 15.9% for bivalent vaccinations, respectively. ≥90% of initial vaccinations occurred in an office practice. In at-risk patients, median time to first-dose received was 11.8, 20.9, and 20.9 months for hepatitis A, hepatitis B, and hepatitis A/B vaccines, respectively.

Primary care and office practices were the most common providers and places of vaccination, respectively, for hepatitis A and B vaccine. For at-risk patients, further research is needed to design vaccination strategies to improve the median time from first ACIP-recommended condition diagnosis to initial vaccination against hepatitis A and B.

## Highlights

• Most frequent provider type for hepatitis vaccination is primary care providers.
• Physician offices are the most frequent site for hepatitis vaccinations.
• Time to initial hepatitis vaccination from first at-risk diagnosis needs improvement.

## 1 Introduction

Hepatitis A and B are viral infections of the liver and are vaccine-preventable. Hepatitis A is transmitted by the fecal-oral route (Matheny et al., 2012) and hepatitis B by percutaneous or mucosal exposure to infectious blood or other body fluids (Mast et al., 2006). The Centers for Disease Control and Prevention (CDC) Surveillance system reported an increase in hepatitis A by 12% and acute hepatitis B by 21%, from 2014 to 2015 among US adults (Centers for Disease Control and Prevention, 2017a). The years 2017–2018 were marked by hepatitis A outbreaks in multiple US states, mainly affecting people who are homeless or illicit drug users (Centers for Disease Control and Prevention, 2017b). In 2015, CDC estimated the number of people suffering from chronic hepatitis B virus (HBV) at 850,000, and the number of new HBV infections, adjusted for under-ascertainment and under-reporting, at 21,900 (95% CI = 12,500–53,600) in the US (Centers for Disease Control and Prevention, 2017a). Acute hepatitis B incidence rate, in the overall population, was 1.1 cases per 100,000 population, 31% of which had at least one high-risk condition diagnosis in the six months before disease onset (Centers for Disease Control and Prevention, 2017a).

Low hepatitis A and B vaccination coverage rates are reported for adults at an increased risk for infection, morbidity, or mortality (Mast et al., 2006; Fiore et al., 2006). For example, in 2015, only 9% of patients ≥19 years of age with chronic liver disease (CLD) had received ≥2 hepatitis A vaccine doses, and 27% had received ≥3 hepatitis B vaccine doses; only 24% of patients with diabetes (19–59 years) and 65% of health care personnel were vaccinated against hepatitis B (Williams et al., 2017). Hepatitis B vaccination coverage of health care personnel, at 64.7%, is well below the 90% target of the Healthy People 2020 objectives (Williams et al., 2017; Office of Disease Prevention and Health Promotion, 2017).

Of the factors that influence an adult's decision to receive a hepatitis A or hepatitis B vaccine, physician recommendation is among the most important (Bridges et al., 2015; Kim et al., 2014; Winston et al., 2006). Thus, identifying provider types and places most commonly associated with the administration of hepatitis vaccinations would provide information on where medical education and resources might enhance vaccine coverage.

This study aimed to provide a descriptive report of the provider type and place of vaccination among adults who received any hepatitis A, B, or A/B vaccine. This study further estimated the time to initial vaccination from the first diagnosis of a condition for which the ACIP recommends hepatitis A and/or hepatitis B vaccination, among an at-risk population, defined based on select ACIP recommendations.

## 2 Methods

### 2.1 Study design and data source

A retrospective cohort study was conducted using data from a large US administrative claims database from Q1 2007 to Q3 2015 (Truven Health Analytics MarketScan) (Hansen and Chang, 2011). The two MarketScan databases analyzed for this study include the Commercial Claims and Encounters database and the Medicare Supplemental and Coordination of Benefits database.

The Commercial Claims and Encounters database provides the enrolment history, medical claims, and pharmacy claims for about 30 million people covered by approximately 100 employers and several health plans from all census regions. The Medicare Supplemental and Coordination of Benefits database focuses on patients aged 65 years and older with Medicare coverage plus employer-paid commercial plans (Hansen and Chang, 2011).

### 2.2 Setting

The period from the date of health plan enrolment to the index date, inclusive was the “baseline period” and was required to be ≥12 months. All patients' diagnoses included in the present analysis were made during this baseline period.

Index date was the date of the first claim with a Current Procedural Terminology (CPT) code corresponding to one of the following three vaccine types: a) a monovalent hepatitis A vaccine, b) a monovalent hepatitis B vaccine, or c) a bivalent hepatitis A/B vaccine.

### 2.3 Participants

Eligible patients had 1) ≥1 claim for a hepatitis A, B, or A/B vaccine, 2) ≥19 years of age at first claim (index date), 3) ≥12 months of continuous health insurance pre-index date (baseline period), and 4) ≥18 months of continuous health insurance post-index date. The latter eligibility criterion served to evaluate vaccination series completion. However, this part of the analysis has been published elsewhere (Ghaswalla et al., 2018). A post-hoc sensitivity analysis was also conducted to describe the provider type and place of hepatitis A, B, or A/B vaccination among patients who met eligibility criteria 1) and 2) only, to ensure robustness of this analysis. This is because patients who may have intermittent insurance coverage would have been excluded by the continuous enrollment criteria in 3) and 4).

For the time to initial vaccination analysis of the at-risk population (referred to here as the “ACIP recommended group”), we excluded patients with a history of hepatitis A or hepatitis B prior to an associated hepatitis vaccine. Two additional selection criteria were therefore applied for this at-risk population: a) <2 diagnostic claims for hepatitis A or B during baseline period, and b) ≥1 diagnostic claims for a select ACIP recommended condition. The select ACIP recommended conditions used in this study were: a) acute and chronic hepatitis B or C, CLD, clotting-factor disease, high-risk sexual behavior, or illicit drug use, for the hepatitis A cohort and b) acute and chronic hepatitis C, chronic kidney disease, CLD, diabetes, dialysis, end-stage renal disease, high-risk sexual behavior, human immunodeficiency virus, illicit drug use, sexually transmitted disease, and pregnancy, for the hepatitis B cohort. The conditions were identified using the International Classification of Disease, Ninth Revision, Clinical Modification (ICD-9-CM) and CPT codes in claims data (Supplementary Table 1).

Additional post-hoc, subset analysis was conducted to estimate median time to hepatitis B vaccination from first diagnosis of one selected ACIP recommended condition, i.e. diabetes. This condition was selected because during the study period (2011), the ACIP issued a recommendation for hepatitis B vaccine administration as soon as possible after a diabetes diagnosis (Centers for Disease Control and Prevention, 2011). For diabetes patients, the median time to vaccination was determined separately for patients with a first diabetes diagnosis claim during and prior to 2011 (diabetes pre-recommendation) and for those with a first diabetes diagnosis claim after this date (diabetes post-recommendation).

### 2.4 Variables

Baseline characteristics included patient demographics (age, gender, and health plan type) and clinical characteristics (diagnoses, and year of first hepatitis vaccination). Demographic characteristics were determined on the index date, and patients' diagnoses were identified during the baseline period before the index date. Provider type and place of vaccination were documented from the claim on the index date (Supplementary Tables 2–3).

### 2.5 Statistical analyses

Statistics were descriptive. Analyses were presented using frequency distributions for the primary outcomes of provider type and place of vaccination, and for patient baseline characteristics. Survival analysis was used to estimate the median time in months from first diagnosis claim for a condition in select hepatitis A- or B- ACIP recommended group in the baseline period to first vaccination dose. Kaplan Meier curves were used to illustrate time to initial vaccination.

## 3 Results

### 3.1 Participants

In our dataset, 1,544,556 patients received their first hepatitis vaccination at or after reaching 19 years of age. Of those, 22.7% (350,973) were eligible for inclusion in the overall eligible population (Supplementary Fig. 1). In the overall eligible population, 183,326 (52.2%) initiated a hepatitis A vaccine (hepatitis A cohort), 148,119 (42.2%) initiated a hepatitis B vaccine (hepatitis B cohort), and 64,953 (18.5%) initiated a bivalent vaccine (hepatitis A/B cohort). One in four of the overall eligible population (24.8%, 87,186/350,973) were in the ACIP recommended group, where 12,691 patients initiated a hepatitis A vaccine, 57,098 initiated hepatitis B vaccine and 22,456 initiated a bivalent vaccine.

### 3.2 Baseline descriptive data

In the overall eligible population, the mean ages across cohorts were 42.1–45.8 years, and 55.3–60.0% were women. The most common health plan was a preferred provider organization (49.8–56.4%), followed by a health maintenance organization (18.2–21.1%). In the ACIP recommended group, patients were older on average than in the overall eligible group; mean age across cohorts were 47.3–49.1 years.

### 3.3 Provider type and place of vaccination

**Fig. 1:**
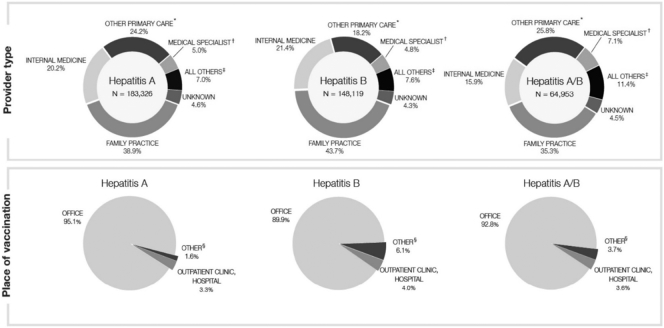
Provider type and place of vaccination, in the overall eligible population, by type of hepatitis vaccine (hepatitis A, hepatitis B, and hepatitis A/B), 2007–2015^1^ ^1^Patients who initiated two monovalent vaccine series were included in both cohorts, hepatitis A and hepatitis B. ^⁎^‘Other primary care’ includes general pediatrics, obstetrics & gynecology, and other primary care physicians; ^†^‘Medical specialist’ includes infectious diseases, gastroenterology, pediatrics, endocrinology, nephrology, and all other specialists; ^‡^‘All others’ includes surgery and surgical specialties, nurse practitioners, nurses, public health agency, pharmacists, and all other health care providers; ^§^‘Other’ includes hospital, pharmacy, end stage renal disease facility, and all others. NOTE: Percentages might not add up to 100% due to rounding.

The main vaccine providers were family practice and internal medicine physicians: 38.9% and 20.2% for hepatitis A, 43.7% and 21.4% for hepatitis B, 35.3% and 15.9% for bivalent vaccinations (Fig. 1). The frequency of vaccination by medical specialists was low (4.8–7.1%). Most patients received vaccinations in an office practice (89.9–95.1%) (Fig. 1). Hospital outpatient clinics were the second most common vaccination setting, accounting for 3.3–4.0% of vaccinations. In the post-hoc sensitivity analysis of the 1,544,556 patients who received their first hepatitis vaccination at or after reaching 19 years of age, similar results were observed, i.e., primary care providers and physician offices were the most frequent provider type and sites of vaccination, respectively (results not shown).

### 3.4 Time to first vaccination in the ACIP recommended group

#### 3.4.1 Hepatitis A and hepatitis B cohorts

**Fig. 2:**
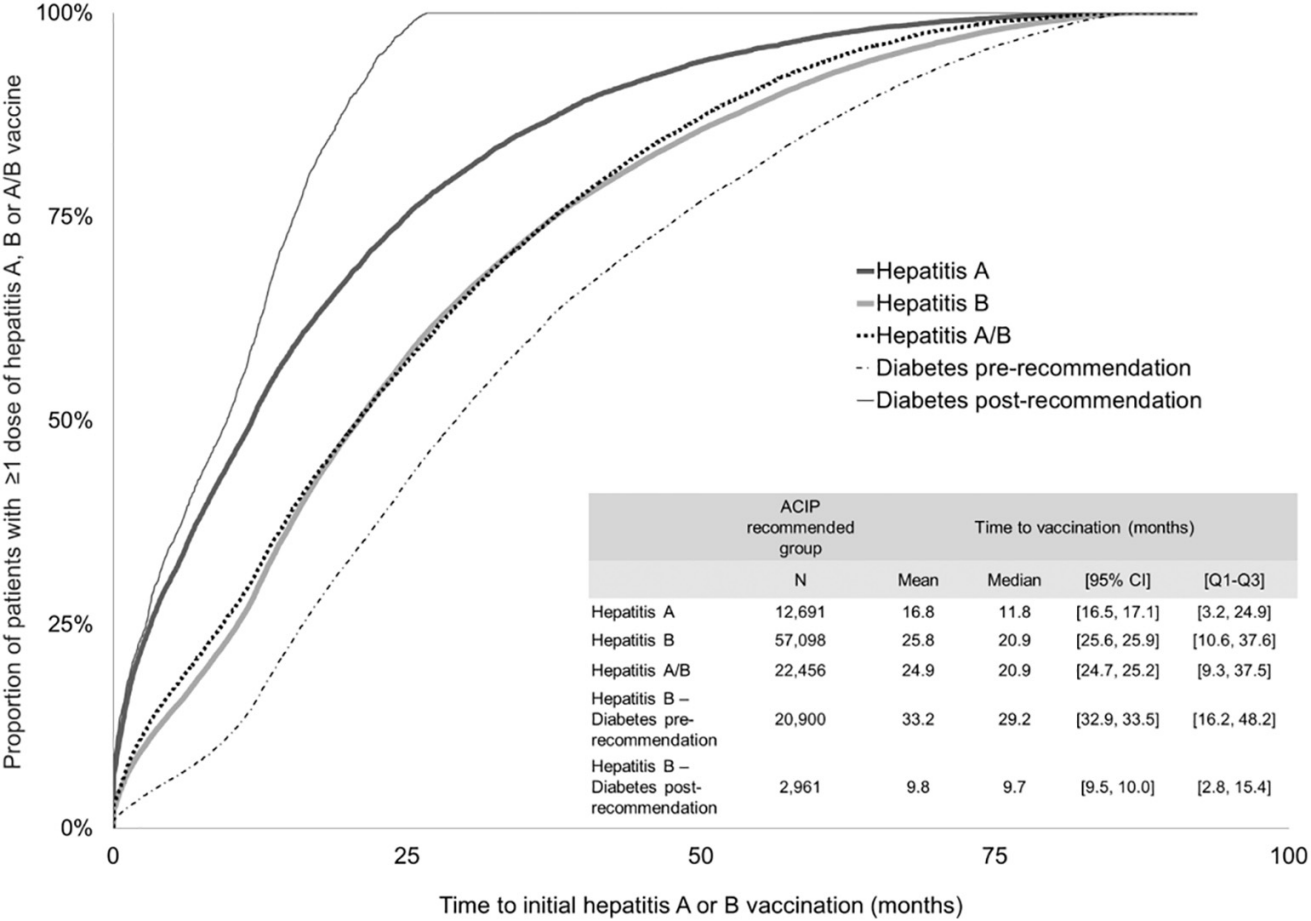
Time from first ACIP recommended condition diagnosis^⁎^ to initial hepatitis A, hepatitis B and hepatitis A/B vaccination, and time from diabetes diagnosis† to initial hepatitis B vaccination, 2007–2015. ⁎The diagnosis of select ACIP recommended conditions took place during the baseline period or on the index date i.e. ≥12 months before the index date. The select ACIP recommended conditions used: a) acute and chronic hepatitis B or C, chronic liver disease (CLD), clotting-factor disease, high-risk sexual behavior, or illicit drug use, for the hepatitis A cohort and b) acute and chronic hepatitis C, chronic kidney disease, CLD, diabetes, dialysis, end-stage renal disease, high-risk sexual behavior, human immunodeficiency virus, illicit drug use, sexually transmitted disease, and pregnancy, for the hepatitis B cohort. †For diabetes patients, the median time to hepatitis B vaccination was estimated and stratified by date of diabetes diagnosis (≤ or > of December 23, 2011, when the year diabetes was first included in the ACIP recommended conditions (Centers for Disease Control and Prevention, 2011)). Abbreviations: ACIP, Advisory Committee on Immunization Practices; CI, confidence intervals

The median time between first diagnosis of an ACIP recommended condition and first dose of vaccination was 11.8 months for the hepatitis A cohort and 20.9 months for the hepatitis B and A/B cohorts (Fig. 2).

#### 3.4.2 Hepatitis B cohort: Post-hoc analysis of patients with diabetes

For the patients with diabetes, the post-recommendation median time to initial hepatitis B vaccination (9.7 months) was shorter than that of the pre-recommendation period (29.2 months) (Fig. 2).

## 4 Discussion

Using a large US database for commercial and Medicare claims data, we identified family practice and internal medicine physicians as the most common hepatitis A, B and A/B vaccination providers for adults in the US, for the period 2007–2015. The most common place of hepatitis vaccination was a physician office. In addition, the median time from first ACIP recommended condition diagnosis to initial hepatitis vaccination was estimated to range from 11.8 to 20.9 months depending on the vaccination cohort. The 2011 ACIP recommendation to provide hepatitis B vaccination as soon as possible after a diabetes diagnosis may have had a positive impact by considerably reducing the time to vaccination in patients with diabetes.

To our knowledge, this is the first study to establish the provider type and place of vaccination for hepatitis A, B and A/B among adults in the US and the time to initial vaccination since diagnosis of an at-risk condition. Most studies on vaccination practices have been cross-sectional surveys of attitudes and perceptions towards vaccination, in either specific health care physicians' specialties (MacDougall et al., 2015; Nelson et al., 2017), or the general population (Winston et al., 2006; MacDougall et al., 2015; Armstrong et al., 2001; Bouder et al., 2015). Many surveys have revealed the influential role of primary care healthcare providers' recommendations in the vaccination decision-making process. A survey in six European countries found that most citizens generally relied on a general practitioner for medical advice and believed that they were the most trustworthy source for advice (Bouder et al., 2015). In the present study, the greatest proportion of initial vaccinations was administered by primary care providers, supporting studies showing the pivotal role primary care providers can play in patient vaccination. The median time to vaccination results underscore the importance of increasing awareness among providers of ACIP recommendations for vaccinating adults at increased risk for hepatitis A or B infection. The effectiveness of electronic medical record (EMR) reminders in increasing the uptake of hepatitis B vaccination among patients with diabetes has been demonstrated previously, (Hechter et al., 2019) and these results warrant further research to determine if median time to initial vaccination after initial diagnosis of an ACIP recommended condition could be decreased through effective interventions.

### 4.1 Study limitations and strengths

Several limitations should be noted when interpreting findings of this study. The MarketScan sample population most commonly comes from large employers and these findings may not be generalizable to individuals covered by other insurance programs, such as Medicaid, or to individuals who are self-insured or uninsured (Hansen, 2017). However, due to its large size, it is representative of a wide variety of socioeconomic levels, and as such, our results may provide a broad base for understanding vaccination practices in a large population of insured persons. Furthermore, ACIP recommended conditions were identified using the ICD-9-CM and CPT codes in claims data; some conditions were therefore not recorded if not identifiable using codes in claims data (e.g. international travelers, current or recent injection drug users, men who have sex with men). Finally, the first observed diagnosis claim of a high-risk condition is not necessarily the first diagnosis in the patient's medical history (i.e., incident case). Hence, the conditions in ACIP recommendation groups as identified in this study were not necessarily incident cases, but cases identified during the baseline period.

## Author contributions

Trofa AF, Cheng WY, Duchesneau E, Macheca M, Patterson BJ, Duh MS were involved in the study design. All authors participated in the interpretation of the study results and the development of this manuscript. All authors had full access to the data and gave final approval before submission.

## Appendix A Supplementary data

Supplementary material 1 Image 1
