# Supplementary material for: The role of provider characteristics on the hepatitis A and B vaccination status of adults in the United States during 2007–2015
Source: Prev Med Rep. 2019 Feb 18;14:100833. doi: 10.1016/j.pmedr.2019.100833 (PMC6402425; doi:10.1016/j.pmedr.2019.100833)
Supplement: Supplementary file 1 — Supplementary material 1 [file mmc1.docx]

**Supplementary Figure 1:** Flowchart of sample selection and classification by vaccination type for hepatitis A, hepatitis B, and hepatitis A/B vaccine-recipients included in commercial and Medicare insurers’ data Q1 2007–Q3 2015

**≥1 dose of hepatitis A or hepatitis B vaccination in adulthood**

**1,544,556**

- Hepatitis A: 774,871
- Hepatitis B: 747,892
- Hepatitis A/B: 224,208

**350,973**

- Hepatitis A: 183,326
- Hepatitis B: 148,119
- Hepatitis A/B: 64,953

**Continuous healthcare insurance: ≥12 months before and ≥18 months after index date***

**<2 diagnostic claims for hepatitis A or B, before index date^†^**

**350,240**

- Hepatitis A: 183,000
- Hepatitis B: 147,453
- Hepatitis A/B: 64,870

**ACIP RECOMMENDED GROUP
≥1 diagnostic claims for a condition in one of the select ACIP recommendation groups^‡^**

**87,186**

- Hepatitis A: 12,691
- Hepatitis B: 57,098
- Hepatitis A/B: 22,456

*Index date was the date of the first CPT code for a monovalent hepatitis A vaccine (CPT-90632), a monovalent hepatitis B vaccine (CPT-90740, 90746, 90747), or a bivalent A/B vaccine (CPT-90636); ^†^<2 diagnostic claims of hepatitis A before a hepatitis A or a hepatitis A/B vaccine, or <2 diagnostic claims of hepatitis B before a hepatitis B or A/B vaccine; ‡The diagnosis of select ACIP recommended conditions (Supplementary Table 3) took place during the baseline period or on the index date i.e. within ≥12 months the index date

Abbreviations: ACIP, Advisory Committee on Immunization Practices; CPT, Current Procedural Terminology

**Supplementary Table 1.** ACIP recommendation groups as defined in this study

| **ACIP recommended condition** | **ICD-9 codes** | **CPT codes** |
| --- | --- | --- |
| **Hepatitis A** | | |
| Acute and chronic hepatitis B | 070.2, 070.20, 070.21, 070.22, 070.23, 070.3, 070.30, 070.31, 070.32, 070.33 | - |
| Acute and chronic hepatitis C | 070.41, 070.44, 070.51, 070.54, 070.7, 070.70, 070.71 | - |
| Chronic liver disease | 571 | - |
| Clotting-factor disorder | 286 | - |
| High-risk sexual behavior | V69.2 | - |
| Illicit drug use (injection drugs) | 304.0, 304.1, 304.2, 304.4, 304.5, 304.7, 304.8, 304.9, 305.3, 305.4, 305.5, 305.6, 305.7, 305.9, 648.33, 648.34, 965.01, 965.09, 967.0, 969.6, 969.72, 970.81, E850.0, E850.1, E850.2 | - |
| **Hepatitis B** | | |
| Acute and chronic hepatitis C | 070.41, 070.44, 070.51, 070.54, 070.7, 070.70, 070.71 | - |
| Chronic kidney disease | 585 | - |
| Chronic liver disease | 571 | - |
| Diabetes | 250 | - |
| Dialysis | V56, V45.1, 39.95. 54.98 | 90935, 90937, 90945, 90947, 90989, 90993, 90921, 90925 |
| Diabetes | 250 | - |
| End-stage renal disease | 585.5, 585.6 | - |
| High-risk sexual behavior | V69.2 | - |
|  |  |  |
| Human immunodeficiency virus | 042 | - |
| Illicit drug use (injection drugs) | 304.0, 304.1, 304.2, 304.4, 304.5, 304.7, 304.8, 304.9, 305.3, 305.4, 305.5, 305.6, 305.7, 305.9, 648.33, 648.34, 965.01, 965.09, 967.0, 969.6, 969.72, 970.81, E850.0, E850.1, E850.2 | - |
| Illicit drug use (non-injection drugs) | 304.3, 304.6, 305.2 | - |
| Persons seeking evaluation or treatment for a sexually transmitted disease | 098, 090, 091, 092, 093, 094, 095, 096, 097, 79.4, 054.1, 616.1, 131 |  |
| Pregnancy | V22, V23 | - |
| **Hepatitis A/B** | | |
| Acute and chronic hepatitis C | 070.41, 070.44, 070.51, 070.54, 070.7, 070.70, 070.71 | - |
| Chronic kidney disease | 585 | - |
| Chronic liver disease | 571 | - |
| Clotting-factor disorder | 286 | - |
| Diabetes | 250 | - |
| Dialysis | V56, V45.1, 39.95. 54.98 | 90935, 90937, 90945, 90947, 90989, 90993, 90921, 90925 |
| Diabetes | 250 | - |
| End-stage renal disease | 585.5, 585.6 | - |
| High-risk sexual behavior | V69.2 | - |
| Human immunodeficiency virus | 042 | - |
| Illicit drug use (injection drugs) | 304.0, 304.1, 304.2, 304.4, 304.5, 304.7, 304.8, 304.9, 305.3, 305.4, 305.5, 305.6, 305.7, 305.9, 648.33, 648.34, 965.01, 965.09, 967.0, 969.6, 969.72, 970.81, E850.0, E850.1, E850.2 | - |
| Illicit drug use (non-injection drugs) | 304.3, 304.6, 305.2 | - |
| Persons seeking evaluation or treatment for a sexually transmitted disease | 098, 090, 091, 092, 093, 094, 095, 096, 097, 79.4, 054.1, 616.1, 131 |  |
| Pregnancy | V22, V23 | - |

**Supplementary Table 2.** Categories of provider type of interest and available labels in database

| **Provider type category and subcategory of interest** | **Provider type label in database** |
| --- | --- |
| **Primary care** | |
| Family practice | Family Practice |
| Internal medicine | Internal Medicine |
| Obstetrics and gynecologists | Obstetrics & Gynecology |
| All Others | Acute Care Hospital  Extended Care Facility  Medical Doctor - MD  MultiSpecialty Physician Group  Treatment Center |
| Pediatrics, General | Pediatrician (NEC) |
| **Medical specialist** | |
| Infectious disease | Infectious Disease |
| Nephrology | Nephrology |
| Gastroenterology | Gastroenterology |
| Endocrinology | Endocrinology & Metabolism |
| All others | Allergy & Immunology  Cardiovascular Dis/Cardiology  Dermatology  Geriatric Medicine  Hematology  Neurology  Oncology  Otolaryngology  Palliative Medicine  Pathology  Podiatry  Pulmonary Disease  Renal Dialysis Therapy  Rheumatology  Urology |
| Pediatrics, all specialties | Child Psychiatry  Neonatal-Perinatal Medicine  Pediatric Allergy & Immunology  Pediatric Cardiology  Pediatric Critical Care Med  Pediatric Emergency Medicine  Pediatric Endocrinology  Pediatric Gastroenterology  Pediatric Hematology-Oncology  Pediatric Infectious Diseases  Pediatric Nephrology  Pediatric Orthopedics  Pediatric Pulmonology  Pediatric Rheumatology  Pediatric Specialist  Pediatric Urology  Sports Medicine (Pediatrics) |
| **Surgery & surgical specialties** | Abdominal Surgery  Ambulatory Surgery Centers  Cardiothoracic Surgery  Cardiovascular Surgery  Colon & Rectal Surgery  General Vascular Surgery  Neurological Surgery  Orthopedic Surgery  Pediatric Surgery  Plastic/Maxillofacial Surgery  Surgeon  Surgical Critical Care  Thoracic Surgery  Transplant Surgery |
| **All others/non-physicians** | |
| Pharmacist | Pharmacist  Pharmacy |
| Nurse practitioner | Nurse Practitioner |
| Nurse, All others | Nurse Anesthetist  Nursing Services  Psychiatric Nurse |
| Public Health Agency | Public Health Agency |
| All other health care provider | Acupuncturist  Anesthesiology  Birthing Center  Case Manager  Chemical Depend Treatment Ctr  Chiropractor/DCM  Convalescent Care Facility  Critical Care Medicine  Dental Specialist  Dentist - MD & DDS  Dietitian  Emergency Medicine  Genetics  Health Educator/Agency  Hearing Labs  Home Health Organiz/Agency  Hospice Facility  Hospitalist  Imaging Center  Infirmary  Intermediate Care Facility  Laboratory  Longterm Care (NEC)  Medical Technician  Mental Health Facilities  Mental Health/Chemical Dep NEC  Mental Hlth/Chem Dep Day Care  Midwife  Nuclear Medicine  Ophthalmology  Optician  Optometrist  Osteopathic Medicine  Other Facility (NEC)  Pain Mgmt/Pain Medicine  Physical Medicine & Rehab  Physician Assistant  Preventative Medicine  Proctology  Psychiatry  Psychologist  Radiology  Rehabilitation Facilities  Residential Treatment Center  Special Care Facility (NEC)  Supply Center  Therapists (Alternative)  Therapists (Supportive)  Therapy (Physical)  Transportation  Urgent Care Facility  Vision Center |

**Supplementary Table 3.** Categories of place of vaccination of interest and available labels in database

| **Place of vaccination category and subcategory of interest** | **Place of vaccination label in database** |
| --- | --- |
| **Office** | |
| Office other than Federal Qualified Health Centre | Office |
| Office | Federally Qualified Health Ctr |
| Urgent care center | Urgent Care Facility |
| Department of Defense | Military Treatment Facility |
| **Hospital** | |
| Acute care hospital/Medical center | Inpatient Hospital |
| Emergency room | Emergency Room - Hospital |
| Long term care facility | Hospice  Inpatient Psychiatric Facility |
|  |  |
| **Pharmacy** | |
| Pharmacy | Pharmacy |
| **End-Stage Liver Disease facility** | |
| End-stage liver disease | End-Stage Renal Disease Facility |
| **Outpatient Hospital** | |
| Outpatient hospital | Outpatient Hospital |
| **Others** | |
| All other locations | Ambulance (land)  Birthing Center  Community Mental Health Center  Comprehensive Outpt Rehab Fac  Group Home  Homeless Shelter  Independent Clinic  Independent Laboratory  Mass Immunization Center  Mobile Unit  Non-resident Substance Abuse Facility  Nursing Facility  Other Unlisted Facility  Outpatient (NEC)  Patient Home  Place of Employment-Worksite  Psych Facility Partial Hosp  Psych Residential Treatmnt Ctr  Rural Health Clinic  School  Skilled Nursing Facility  State/Local Public Health Clin  Temporary Lodging  Walk-in Retail Health Clinic |
| Assisted Living Facility | Assisted Living Center |
| Rehabilitation Centre | Residential Subst Abuse Facil |
| All Other Inpatient Facilities | Ambulatory Surgical Center  Comprehensive Inpt Rehab Fac  Custodial Care Facility |
|  |  |
|  |  |
